# Microsecond Pulse I–V Approach to Understanding Defects in High Mobility Bi-layer Oxide Semiconductor Transistor

**DOI:** 10.1038/s41598-017-06613-1

**Published:** 2017-08-15

**Authors:** Hyunsuk Woo, Sanghun Jeon

**Affiliations:** Department of Applied Physics, Korea University, 2511, Sejongro, Sejong, 339-700 Korea

## Abstract

The carrier transport and device instability of amorphous oxide semiconductor devices are influenced by defects that are exponentially distributed in energy, because of amorphous phase channels and front/back interfaces with a large number of sub-gap states. Thus, understanding defects and charge trapping in oxide semiconductor transistors is required for being core device element in reliable production lines. In this paper, we present the transient charging effect, the charge trapping mechanism, and the dynamic charge transport of high-mobility bilayer oxide semiconductor transistors. To this end, we exploited microsecond ramps, pulse ID–VG, transient current, and discharge current analysis methods. The mobility enhancement rate of single HfInZnO (HIZO) and bilayer HfInZnO-InZnO (HIZO-IZO) were 173.8 and 28.8%, respectively, in the charge-trapping-free environment. Transient charge trapping can be classified to temperature insensitive fast charging and thermally activated slow charging with two different trap energies. Insignificant fast transient charging of a bilayer-oxide high-mobility thin film transistor(TFT) can be explained by the low density of sub-gap states in the oxide semiconductor. Understanding defects and transient charging in the oxide semiconductor helps to determine the origin of device instability of oxide TFTs, and finally, to solve this problem.

## Introduction

In active matrix organic light emitting diode (AMOLED) back planes, amorphous and *c*-axis crystalline oxide semiconductor-based thin film transistors (TFTs) with high performance and high reliability are used as switching and driving elements because of their outstanding electrical and stability characteristics, low-temperature processing availability, and large area deposition capability^1–8^. For high pixel-density, high-frame-rate, and large-area display back panels, TFTs should have high transistor mobility (over 10–30 cm^2^/V·s)^8–15^. Among various oxide semiconductors, double-layer heterojunction structures composed of a high-reliability front channel and a high-mobility back channel (or the reverse structure) have been studied to achieve high mobility while easing the trade-offs among mobility, initial threshold voltage, and reliability characteristics^13–21^. Use of heterojunction oxide semiconductors is a cost-effective strategy in comparison to finding new composite materials^22, 23^. On the other hand, amorphous and nano-crystalline oxide semiconductors have inherent defects in their active channels because of some (or a substantial degree of) disordered crystalline structure. In addition, two different materials of gate insulator/semiconductor and passivation layer/semiconductor result in the formation of a front- and back-channel interface^24–30^. Carrier transport in oxide TFTs is dominated by both defects in the bulk and the interface with sub-gap states exponentially located in energy^31–35^. When a flat-band voltage is applied to the oxide TFT, the injected carriers drift to defect in bulk and interface. With a high gate voltage, the carrier is trapped in sub-gap state by the tunneling. This causes transient charge trapping and device instability^36, 37^. These remain fundamental problems for the successful production of high-resolution, high-frame-rate back panels. Therefore, it is necessary to study the effect of defects, which cause charge trapping and device instability. The conventional DC measurement method is limited to long-time trapping and device instability, making it difficult to obtain an overall picture^38–41^.

In our work, we aim to understand defects and investigate both their impact on device performance and the transient charge trapping characteristics of dual active-layer TFTs with HfInZnO (HIZO) front channels and InZnO (IZO) back channels using pulse *I–V* measurements, and provide an accurate method for determining mobility in an environment with minimal charge trapping^42–46^. To this end, we employed microsecond fast ramp *I–V* (μs-FIV), pulse *I–V* (PIV), the transient current method, and discharge current analysis (DCA). The μs-FIV and PIV methods permit relatively fast sweeping and measurement times on the order of μs, unlike the conventional DC *I–V* method^47–54^. Thus, because we employ μs-FIV measurement, the charge trapping phenomenon induced by the defects can be minimized during measurement, enabling us to extract near-intrinsic transistor parameters. In addition, we extracted the sub-gap states of amorphous oxide TFTs with quantified parameters using the DCA method, which is a modified charge pumping method^55, 56^. In the DCA measurement method, we applied a periodic pulse to the gate at a controlled frequency and measured the discharge current, thereby extracting the sub-gap states that are exponentially distributed in energy in the oxide TFT^57–59^. DCA result shows that the bilayer HIZO-IZO oxide TFT has lower sub-gap states in the oxide TFT, then in the single-layer HIZO oxide TFT. We also discuss the model of charging phenomena of single-layer HIZO and bilayer HIZO-IZO oxide TFTs using the transient current measurement method with varying temperature^36, 51, 52^. We observed fast and slow charge-trapping phenomena for both devices and provide a possible model following temperature-insensitive fast charging and thermally activated slow charging at two different trap energies. The various pulsed *I–V* techniques described in this paper are expected to help accurately extract the sub-gap density of states and the transistor parameters, and improve understanding of the impact of charging on oxide TFTs.

## Results

**Figure 1:**
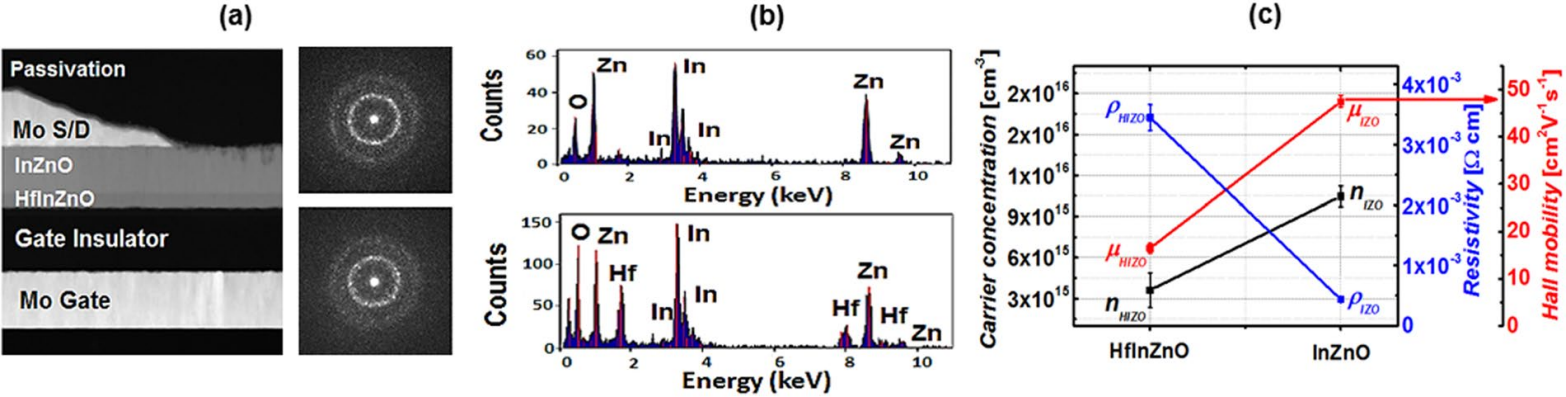
(**a**) Cross-sectional high annular transmission electron microscopy image of bilayer oxide TFT and transmission electron diffraction patterns of IZO (right top) and HIZO (right bottom) (**b**) Energy dispersive spectroscopy data of IZO (top) and HIZO (bottom). (**c**) Comparison of electrical properties for single-layer HIZO and IZO thin film by Hall effect measurement.

**Figure 2:**
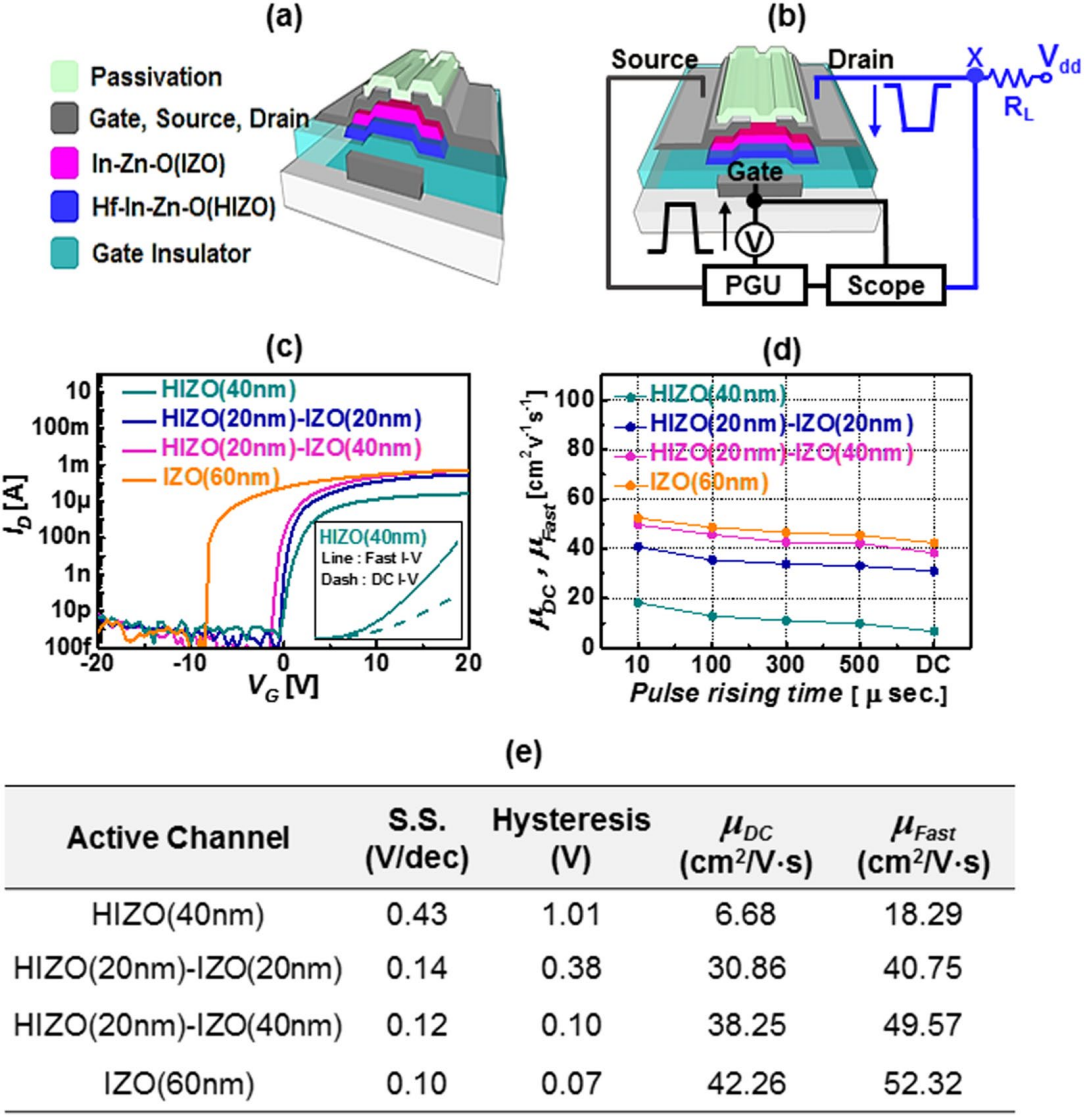
(**a**) Basic schematics of bilayer oxide TFT with bottom gate, top contact, and passivation layer. (**b**) schematic of μs-FIV/PIV system setup. (**c**) DC *I–V* characteristics of various oxide TFTs. Inset shows representative fast *I–V* and DC *I–V* data for HIZO TFT. (**d**) Extracted mobility versus voltage ramping time for various oxide TFTs. (**e**) Sub-threshold slope, hysteresis, and mobility extracted from DC *I–V* and fast *I–V* measurement methods for four different active oxide semiconductor transistors.

As seen in Fig. 1(a), the cross-sectional high-resolution scanning transmission electron microscopy image shows the Mo bottom gate TFT with a top-contact Mo source/drain and passivation layer structure. An inverted staggered bottom-gate oxide TFTs were fabricated using a standard semiconductor fabrication process technology. For all of the electrode, Mo electrode was deposited by radio frequency (RF) sputtering using O_2_/Ar gas and subsequently growing a gate insulator of SiO_2_ by the plasma-enhanced chemical vapor deposition (PECVD). As an active channel, four different type of semiconductor layer configurations, such as HIZO (40 nm), HIZO (20 nm)-IZO (20 nm), HIZO (20 nm)-IZO (40 nm) and IZO (60 nm) channel, were employed. Each active channel was deposited at room temperature by RF magnetron sputtering method. Subsequently, source/drain electrodes were sputtered at room temperature, followed by pattering process. Then PECVD SiO_x_ passivation layer was formed and contact hole was patterned. The detail of fabrication can be found in the Methods (Device Fabrication). The transmission electron diffraction pattern images of the bilayer semiconductors, top IZO, and bottom HIZO, in the right part of Fig. 1(a), show tiny dots and characteristics lines in the hazy background image, implying that both semiconductor layers are composed of the nano-crystalline phase in an amorphous medium^4^. The energy dispersive spectroscopy data in Fig. 1(b) verifies that top and bottom layers are made of IZO and HIZO, respectively. For composition analysis, the concentrations of Hf, In, and Zn were determined from different HIZO and IZO samples using inductively coupled plasma atomic emission spectrometry (ICP-AES) on a Shimadzu ICPS-8100 sequential spectrometer. We found that for IZO, Zn/(In + Zn) and In/(In + Zn) are 0.51 and 0.49, respectively. For HIZO, Zn/(Hf + In + Zn), In/(Hf + In + Zn), and Hf/(Hf + In + Zn) are 0.4, 0.53, and 0.07. For electrical analysis on both samples, the Hall mobility and carrier concentration of HIZO and IZO were determined using Hall effect measurement (HIZO; μ: 17.2 cm^2^/V·s, *n*_carrier_: 2.71 × 10^15^ cm^−3^ and IZO; μ: 48.4 cm^2^/V·s, *n*_carrier_: 9.93 × 10^15^ cm^−3^), as shown in Fig. 1(c). In our study, the front HIZO semiconductor layer adjusts the threshold voltage and the back IZO semiconductor layer was designed as a high-mobility channel^10, 60, 61^. A schematic of the bilayer oxide TFT is presented in Fig. 2(a). For μs-FIV, PIV, and DCA measurements, we designed the device such that it minimized the overlap capacitances among the gate, source, and drain. Thus, all electrodes were patterned using conventional lithography and dry etching methods. A schematic of the μs-FIV/PIV measurement is shown in Fig. 2(b). For fast-pulse electrical testing, we used the waveform generator/fast measurement unit (WGFMU) module in the B1500A semiconductor parameter analyzer. For the DC *I–V* measurements, the voltage sweep rate was 1 V/s; for μs-FIV and PIV measurements, a single pulse (rise time and fall time 10 μs, pulse width 2 ms) was used. DCA was performed using a high-speed pulse generator (Agilent 81104 A) and a source meter (Keithley 2401) to obtain the quantitative parameters of the number of sub-gap states in the oxide TFT.

**Figure 3:**
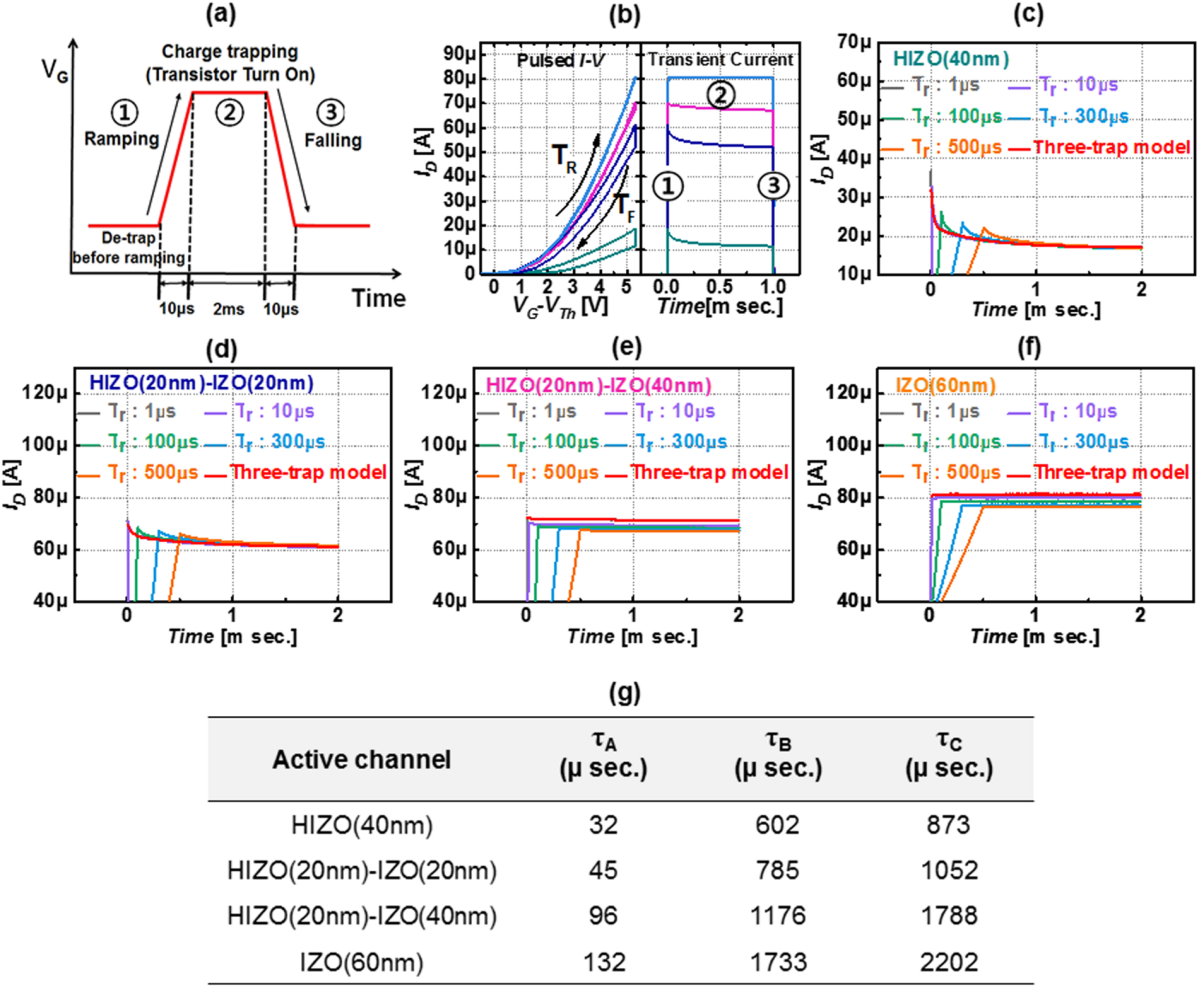
(**a**) Voltage ramp profile of single pulse measurements (rising time = falling time = 10 μs and pulse width = 2 ms). (**b**) (Left figure) Single pulse *I–V* data and (right figure) corresponding transient current versus time data for four oxide TFTs. (**c**–**f**) Transient current data of HIZO (40 nm), HIZO (20 nm)-IZO (20 nm), HIZO (20 nm)-IZO (40 nm) and IZO (60 nm) as a function of rising time and fitted using the three-trap model. (**g**) Charge trapping time constant extracted from the fitting curve the multiple-trapping model.

Figure 2(c) shows a typical DC *I–V* curve of four TFTs with active layers of HIZO (40 nm), HIZO (20 nm)-IZO (20 nm), HIZO (20 nm)-IZO (40 nm) and IZO (60 nm). When a relatively thick IZO layer is used, the drain current level is increased because of both the high carrier concentration and the high mobility of IZO as the IZO thickness increases^60, 61^. The DC *I–V* measurement method has a relatively long sweep/measurement time, and thus, the tested device experiences significant electrical stresses, thereby leading to significant charge trapping, mobility degradation, and device instability^42^. Therefore, we employed a fast *I–V* measurement method to minimize the fast trapping effect, thereby improving drain current and mobility. Properties were measured at a relatively rapid rise rate of several microseconds, as compared to conventional DC *I–V*. As shown in the inset in Fig. 2(c), the fast *I–V* measurement technique for the HIZO TFT compensates for the fast trapping effects, showing a higher drain current than the DC technique. Figure 2(d) shows the mobility versus voltage ramping time (i.e., 10 μs, 100 μs, 300 μs, and 500 μs). As expected, with shorter voltage ramping times, there is higher drain current because of minor charge trapping. Charge trapping depends on gate voltage ramping time. Slow voltage ramping results in high charge trapping. Such a microsecond short sweeping and measurement time can avoid and minimize some channel carriers to be trapped in the shallow-level defects placed in the semiconductor with a certain level of sub-gap states also gate insulator film or at the interface. Therefore, a fast I-V measurement method has been employed to minimize the effect of fast charging, leading to evaluate the near-intrinsic mobility. Various mobility values of the four devices are presented Results show that the highest mobility was achieved with bilayer HIZO-IZO devices with relatively thick IZO layers. In addition, the mobility increase rates (*μ*_fast_/*μ*_dc_) for HIZO (40 nm), HIZO (20 nm)-IZO (20 nm), HIZO (20 nm)-IZO (40 nm) and IZO (60 nm) TFTs were 173.8%, 38.5%, 28.8% and 23.8% respectively, indicating that bilayer devices with relatively thick IZO layers have fewer defects. As will be discussed below in detail, this phenomenon occurs because HIZO has a relatively large number of acceptor-like sub-gap states, causing charge trapping and device instability^62–65^. To further analyze the transient charging effect, we performed single-pulse *I–V* measurements. The voltage ramping profile for this measurement is shown in Fig. 3(a). In the single-pulse *I–V* measurement setup, we started to apply a relatively negative bias to the gate. During this stage, residual charges, which might be trapped in defects, are discharged. In the first stage, we ramp the gate voltage from 0 V to the transistor turn-on voltage during a rise time of 10 μs and simultaneously measure the drain current. In the second step, the transistor is turned on and some of the majority channel carriers might be trapped in the defects, causing a shift of the threshold voltage toward the positive gate bias direction and reducing the drain current level, because we applied the pulse for 2 ms and the drain current was gradually reduced during this time because of charge trapping. In the third phase of single-pulse measurement, we ramped down the gate voltage from the transistor turn-on voltage to 0 V during a fall time of 10 μs while measuring the drain current. For the three steps, the measured drain current versus gate voltage plots for four devices during rising and falling times (T_R_ and T_F_, respectively) are presented in the left part of Fig. 3(b). During single-pulse measurement (composed of three phases), the transient drain current versus pulse time for four devices was measured; results are plotted in the right part of Fig. 3(b). When we performed singe-pulse measurement at various voltage rising times (i.e., 1 μs, 10 μs, 100 μs, 300 μs, and 500 μs), the transient drain current during the pulse time gradually becomes saturated after 0.5 ms, as seen in Fig. 3(c–f), following typical charge trapping behavior. The reduction of drain current during the pulse indicates charge trapping, and that transient charging behavior is significant for a single-layer HIZO TFT, but less so for a bilayer oxide TFT with relatively thin IZO, and insignificant for bilayer oxide TFT with relatively thick IZO, which is consistent with the result presented in Fig. 2. Transient charging behavior for four devices match the three-trap model with three trap time constants, as given by Eq. (1)^43^.

1 $${\rm{I}}={\rm{A}}{I}_{0}\exp (-\frac{t}{{\tau }_{A}})+{\rm{B}}{I}_{0}\exp (-\frac{t}{{\tau }_{B}})+{\rm{C}}{I}_{0}\exp (-\frac{t}{{\tau }_{C}})$$

The above equation gives the number of events. The trapping time constants for the four oxide TFT devices are shown in Fig. 3(g). A short capture time constant (**τ**_**A**_) and a long capture time constant (**τ**_**B**_ and **τ**_**C**_) were extracted from Fig. 3(c–f) by applying the above equation. This result reveals that with quantified constants, charge trapping phenomena during charge transport through the channel are significantly affected by the active structure^43, 45^. Generally, carrier transport and charge trapping are thought to occur mainly between the channel and the insulator, or injected into the insulator from the channel^50–54, 66–68^. However, in this study, we found that the effect of the IZO back channel is pronounced in TFTs with the same front channel (HIZO) and the same front interface (SiO_2_-HIZO). Because the back channel IZO layer has high mobility and high carrier concentration, this layer is attributed to the main charge transport layer in the HIZO-IZO stacked oxide TFTs. When charge transport occurs via an IZO layer with relatively low sub-gap states, charge trapping phenomena occur less often.

**Figure 4:**
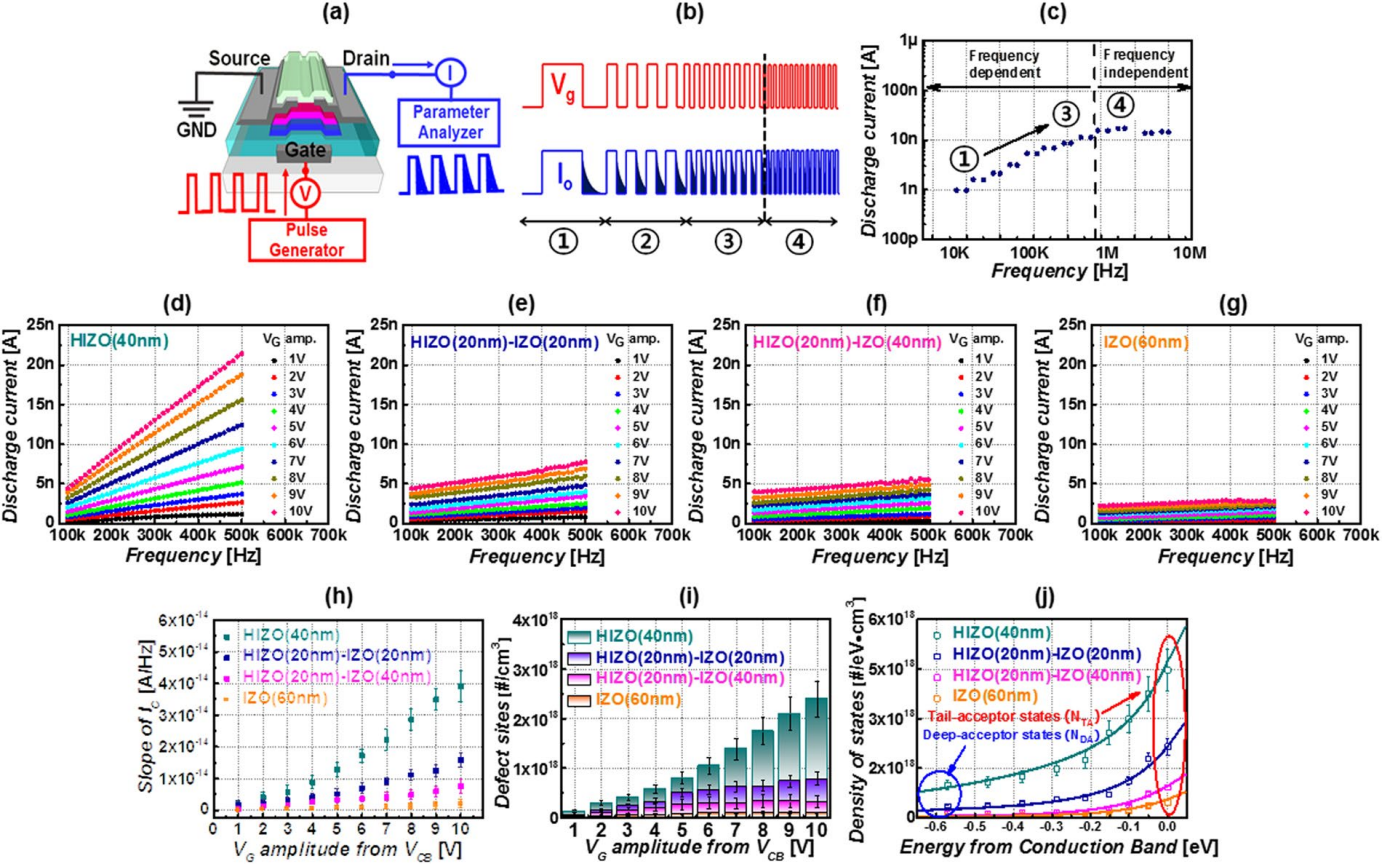
(**a**) Overview of discharge current analysis methods. (**b**) Overview of voltage pulse profile and discharge current versus measurement frequency. (**c**) Discharge current data versus measurement frequency. (**d**–**g**) Discharge current versus pulse frequency for TFTs with four different active channels, i.e., HIZO (40 nm), HIZO (20 nm)-IZO (20 nm), HIZO (20 nm)-IZO (40 nm) and IZO (60 nm) as a function of voltage profile. (**h**) The slope of discharge current versus frequency for four oxide TFTs as a function of voltage. (**i**) Defect densities versus gate voltage for the four oxide TFTs. (**j**) Density of states versus energy for the four oxide TFTs.

To extract the sub-gap states of the oxide TFT of four different channel structures, we exploited a DCA method modified by the charge pumping method^55–59^. Figure 4(a) shows the measurement diagram of the DCA method. The pulse generator (Agilent 81104 A) and the source meter (Keithley 2401) were controlled using the Lab-view program. We applied a periodic voltage pulse to the gate while the end nodes of source and drain were grounded, enabling us to modulate the carrier concentration. Figure 4(b) shows the gate pulse shape and corresponding discharge current versus the measurement frequency. When we ramp up the gate voltage, the interface and the bulk channel (with and without sub-gap states) are charged. Upon ramping down the gate voltage, the majority carriers in the channel are quickly discharged via source and drain. Thereafter, charges trapped in the interface and the channel with sub-gap states are slowly discharged. At a certain read time, we measured this discharge current at the drain using the source meter. Therefore, the tail part of the discharge current, δ*I*, is related to the carriers discharged from the interface and the bulk channel with sub-gap states. The details of the measurement method and related physics are found in previous work^57–59^. As we measure the discharge current as a function of measurement frequency for the oxide TFT Fig. 4(b and c), the discharge current level varies with measurement frequency. At measurement frequency changes from 10 kHz to 1 MHz (from the 1^st^ to the 3^rd^ step), the discharge current linearly increases. Then, above 1 MHz, the value becomes saturated. For extracting the number of defects, we measured the discharge current of the device at a measurement frequency of 100–500 kHz. The number of charging sites is extracted from the slope, δ*I*/δ*f*, using Eq. (2),

2 $${N}_{Defectsites}[\#/c{m}^{2}]=\frac{2}{k}\cdot \frac{{\rm{\delta }}I/{\rm{\delta }}f}{V\cdot q}\,[\frac{(C/t)\cdot t}{c{m}^{3}\cdot C}]$$

where *N*_*Defect sites*_ is the density of defects (sub-gap states), *k* is the charge loss factor, and *V* is the active volume^57^. Additionally, we measure the measurement-frequency-dependent discharge current as a function of gate pulse amplitude (V_G amp_ = V_G_ − V_FB_). As shown in Fig. 4(d–g), the discharge current and the slope, δ*I*/δ*f*, of the single-layer HIZO TFT is sharp, but is relatively lower for the bilayer oxide TFT with increasing IZO thickness. As shown in Fig. 4(h–j), from the slope, δ*I*/δ*f*, of the oxide TFTs, the density of the sub-gap states was extracted. The energy distribution of *N*_*Defect sites*_ [*N*_*Defect sites*_ (E)] is obtained by calculating the surface potential *ϕ*_*s*_ (or *E*) as a function of *V*_*G*_ using Eq. (3)^59^.

3 $${\varphi }_{s}({V}_{GS})={\int }_{{V}_{FB}}^{{V}_{GS}}(1-\frac{{C}_{G}({V}_{GS})}{{C}_{i}})d{V}_{GS}$$

Figure 4(j) presents the sub-gap density profile of exponentially distributed in energy. The density of the sub-gap states in the four oxide devices, namely, HIZO (40 nm), HIZO (20 nm)-IZO (20 nm), HIZO (20 nm)- IZO (40 nm) and IZO (60 nm) TFTs are 1.2 × 10^18^, 3.7 × 10^17^, 1.6 × 10^17^, 8.6 × 10^16^/cm^3^, respectively. Table 1 summarizes the parameters of the sub-gap density profile from the model *g*(*E*) = *N*_*DA*_·*exp*{(*E* − *E*_*C*_)/*kT*_*DA*_} + *N*_*TA*_·*exp*{(*E* − *E*_*C*_)/*kT*_*TA*_}, where *N*_DA_ is the acceptor-like deep state density, *kT*_DA_ is the acceptor-like deep state characteristic energy, *N*_TA_ is the acceptor-like tail state density, and *kT*_TA_ is the acceptor-like tail state characteristic energy^62–65, 69^.

**Table 1:** Parameters of sub-gap density of states extracted using the discharging current analysis method.

Active channel	*N*_*TA*_ (cm^−3^ eV^−1^)	*kT*_*TA*_ (eV)	*N*_*DA*_ (cm^−3^ eV^−1^)	*kT*_*DA*_ (eV)
HIZO (40 nm)	6.1 × 10^18^	0.09	7.4 × 10^17^	0.5
HIZO (20 nm)-IZO (20 nm)	2.3 × 10^18^	0.11	3.5 × 10^17^	0.6
HIZO (20 nm)-IZO (40 nm)	1.2 × 10^18^	0.12	1.1 × 10^17^	0.6
IZO (60 nm)	8.5 × 10^17^	0.14	1.0 × 10^17^	0.6

**Figure 5:**
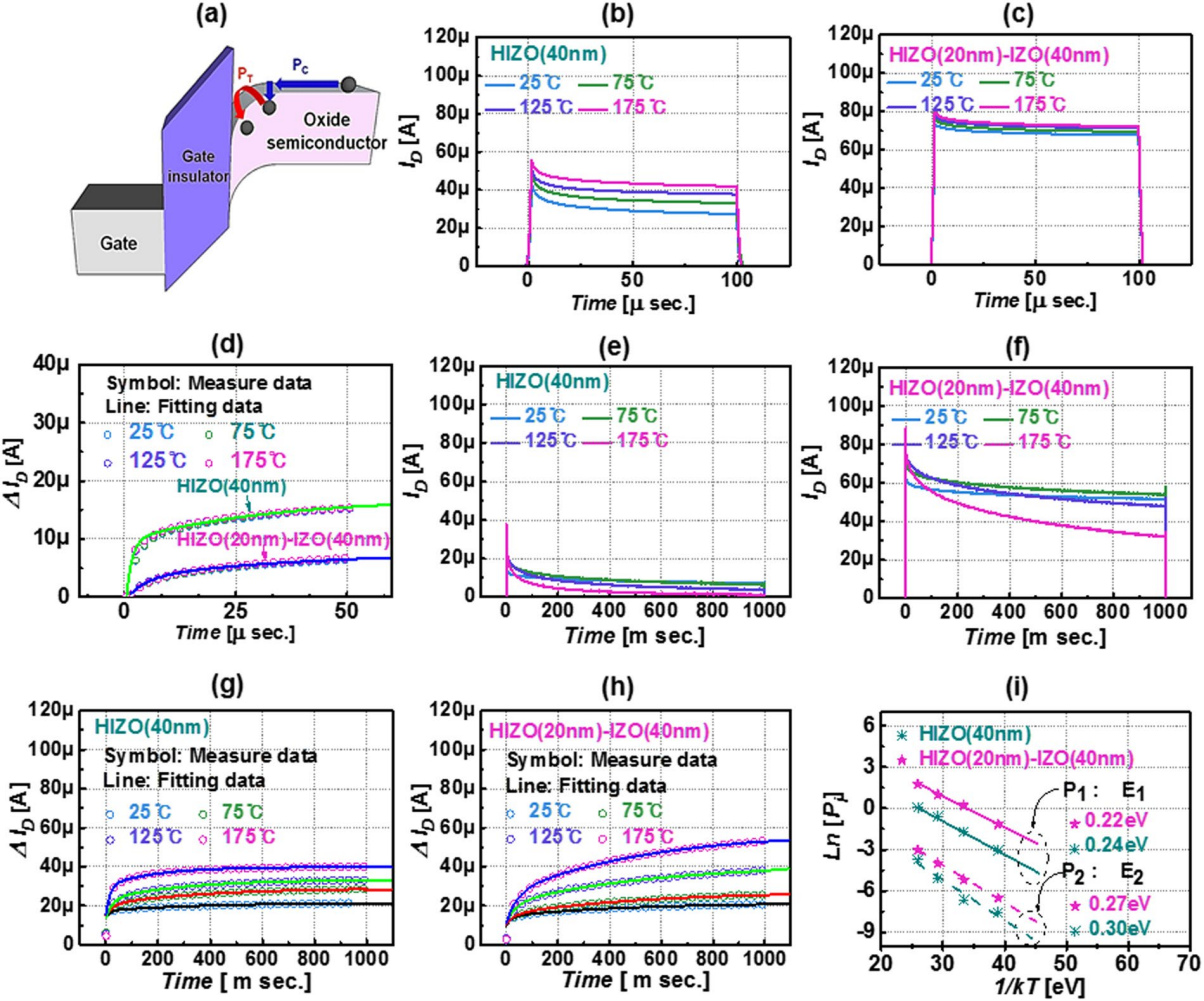
(**a**) Transient charge trapping model in oxide semiconductor TFT. (**b**,**c**) Representative transient current data of HIZO and HIZO-IZO oxide TFTs at different measurement temperatures (25 to 175 °C) during the short pulse of 100 μs. (**d**) Experimental (symbol) and modeled (line) drain current change for 50-μs short pulse time for both devices. Transient current data of (**e**) HIZO and (**f**) HIZO-IZO oxide TFTs during the relatively long pulse time (1000 ms). Experimental (symbol) and modeled (line) transient current reduction of (**g**) HIZO and (**h**) HIZO-IZO oxide TFTs. (**i**) Activation energies *E*_*i*_ extracted from charge trapping probability, *p*_*i*_ values versus measurement temperature for both oxide TFTs.

To further understand the transient charge trapping phenomena, the transient drain current versus pulse time plots for representative single-layer HIZO and bilayer HIZO-IZO TFTs were measured with different measurement temperature, as shown in Fig. 5. We used the model of G. Bersuker in which the existing sub-gap states or defects are filled via two processes, as shown in Fig. 5(a) ^36, 51, 52^. As we apply a positive gate voltage, electrons drifting toward the front active channel are charged or trapped by acceptor-like sub-gap states. This step is called P_C_, which is a dominant process in the fast-transient charge trapping mechanism. Then, when electrons have sufficient thermal energy, the charged or trapped electrons are activated to overcome the barrier, then move via the trap (process P_T_). On the other hand, P_T_ is a secondary (and slow) transient electron trapping process based on electrons having sufficient thermal energy^36^. In this model, the equation for the kinetics of electron trapping can be expressed as follows.

4 $$n={N}_{0}(1-{e}^{-pt})$$

Here, *n* is the number of the occupied defect-sites, *N*_*0*_ is the total available trap number, *p* is the electron-trapping probability, and *t* is the time for the process. Figure 5(b and c) show the transient drain current of HIZO and HIZO-IZO layers versus measurement temperature. By fitting equation (4) to Δ*I*_*D*_ during the short charging time (initial 50 μs), we analyzed fast electron trapping characteristics, which indicate that fast charging is not dependent on measurement temperature. For single-layer HIZO and bilayer HIZO-IZO TFTs, the extracted *N*_0_ values are on the order of 8.4 × 10^13^ and 2.2 × 10^12^ cm^−2^, respectively.

To probe the slow transient charge trapping phenomena, the transient drain current versus time was obtained during the long stress time (approximately 1000 ms), as depicted in Fig. 5(e,f), because slow transient charging follows a thermally activated process from trap to trap via activation energy. According to this model including the thermal activation process, the kinetics of the charging process can be expressed by Eq. (5).

5 $${{\rm{J}}}_{{\rm{s}}}({\rm{i}})={\rm{n}}\frac{1}{{\rm{\tau }}}\exp (-\frac{{{\rm{E}}}_{{\rm{i}}}}{{\rm{kT}}})$$

where *J* is the current density, τ is the de-trapping time constant, *E*_*i*_ is the trap energy, *k* is the Boltzmann constant, and *T* is temperature. Then, the slow transient charge trapping process with time is given by Eq. (6).

6 $${\rm{N}}={{\rm{N}}}_{{\rm{s}}}\sum _{{\rm{i}}}(1-{{\rm{e}}}^{-{{\rm{p}}}_{{\rm{i}}}{\rm{t}}})$$

In our study, we used two terms in Eq. (6) based on fitting for all measurement temperatures. Figure 5(g and h) show measured (symbol) and fitting (line) *ΔI*_*D*_ data for the slow transient charging model for two devices, respectively. The relation of [ln(*P*_*i*_) versus 1/*kT*] generates *E*_1_ and *E*_2_, which represent trap energy barriers for slow transient charge flux: *E*_1_ = 0.24 eV, *E*_2_ = 0.30 eV (single-layer HIZO) and *E*_1_ = 0.22 eV, *E*_2_ = 0.27 eV (bilayer HIZO-IZO), respectively, as shown in Fig. 5(i). This result indicates that the single-layer HIZO TFT has a larger number of defects and is more vulnerable to transient charge trapping, relative to the bilayer HIZO-IZO TFT.

## Discussion

In summary, we evaluated the transient charge trapping effect, the charge trapping mechanism, and the dynamic charge transport of single-layer HIZO and high-mobility bilayer HIZO-IZO oxide TFTs. In our study, we carried out μs-FIV, PIV, transient current, and DCA analysis methods. The results indicate that the mobility enhancement rate of single-layer HIZO and bilayer HIZO-IZO is closely related to the charge trapping effect. The charge trapping mechanism follows the three-trap model with three different trapping time constants. The transient charge trapping model for both TFTs follows temperature-insensitive fast-transient charging and thermally activated slow transient charging with shallow and deep trap energies. For single-layer HIZO and bilayer HIZO-IZO TFTs, the extracted *N*_0_ values are on the order of 8.4 × 10^13^ and 2.2 × 10^12^ cm^−2^, respectively. Insignificant fast-transient charging of the bilayer HIZO-IZO TFT can be explained by the low density of sub-gap states in oxide TFTs. Various trap analysis methods allow us to evaluate defects; transient charge trapping study of oxide semiconductors improves understanding of the device instability and contributes to solving this problem.

## Methods

### Device Fabrication

We fabricated a bilayer oxide semiconductor TFT using an inverted staggered structure, as shown in Fig. 2(a). The integration of metal oxide semiconductor bilayer TFTs was done by sputter deposition a 100-nm thick Mo electrode at room temperature and subsequently growing a gate insulator of 100-nm thick SiO_2_ by the plasma-enhanced chemical vapor deposition (PECVD). As an active channel, four different type of semiconductor layer configurations (such as HIZO (40 nm), HIZO (20 nm)-IZO (20 nm), HIZO (20 nm)-IZO (40 nm) and IZO (60 nm) channel) were employed. The films were deposited at room temperature by radio frequency (RF) magnetron sputtering method using separate 4inch diameter target of In_2_O_3_, ZnO, and HfO_2_. The RF power supplied to each of the targets was adjusted to control the composition of HIZO and IZO. The composition of the channel was analyzed by ICP-AES. The cation ratio was Hf/In/Zn = 0.07/0.53/0.4 for HIZO channel and In/Zn = 0.49/0.51 for the IZO channel. Subsequently, a 100-nm thick source/drain electrodes were sputtered at room temperature, followed by pattering process. Then, 200-nm thick PECVD SiO_x_ passivation layer was formed and contact hole was patterned. After fabrication, the TFT devices were subjected to a post-annealing process in air for 2 h at 200 °C to cure any possible defects in the TFTs.

### Device Characterization Device Characterization

The DC *I-V*, μs-FIV, and PIV transient current methods were applied using the Agilent waveform generator fast measurement unit (WGFMU) module of the B1500A semiconductor device analyzer. The WGFMU module creates pulses and arbitrary linear waveforms. These are applied to the device via the remote sensing unit (RSU) and current measurements are made using the RSU connected to the drain terminal. For the DC *I*–*V* measurements, the voltage sweep rate was 2 V/s, whereas for the μs-FIV and PIV measurements, the voltage scan rate was 1 V/µs. For DCA, square pulse bias in 100 kHz to 500 kHz range was applied to the gate electrode using a pulse generator (Agilent 81104 A). The pulse width was determined by the duty cycle of the pulse (50%) at a given frequency; the rising/falling time was fixed at 80 ns. The source is connected to ground within the source meter; the drain is connected to the parameter analyzer (Keithley 2401).

## References

[1] Kwon JY (2015). Recent progress in high performance and reliable n-type transition metal oxide-based thin film transistors. Semicond. Sci. Technol.

[2] Nomura K (2004). Room-temperature fabrication of transparent flexible thin-film transistors using amorphous oxide semiconductors. Nature.

[3] Jeong JK (2013). Photo-bias Instability of Metal Oxide Thin Film Transistors for Advanced Active Matrix Displays. J. Mater. Res.

[4] Jeon S (2014). Origin of High Photoconductive Gain in Fully Transparent Heterojunction Nano-crystalline Oxide Image Sensors and Interconnects. Advanced Materials.

[5] Nathan A, Lee S, Jeon S, Robertson J (2014). Amorphous Oxide Semiconductor TFTs for Displays and Imaging. Journal of Display Technology.

[6] Lee S, Jeon S, Chaji R, Nathan A (2015). Transparent Semi conducting Oxide Technology for Touch Free Interactive Flexible Display. Proc. IEEE.

[7] Jeong JK (2008). Origin of Threshold voltage instability in Indium-Gallium-Zinc Oxide Thin Film Transistors. Appl. Phys. Lett.

[8] Park J-S (2008). Novel ZrInZnO Thin-film Transistor with Excellent Stability. Advanced Materials.

[9] Van de Walle CG (2000). Hydrogen as a cause of doping in zinc oxide. Phys. Rev. Lett..

[10] Choi H-S (2011). Influence of Hf contents on interface state properties in *a*-HfInZnO thin-film transistors with SiN_x_/SiO_x_ gate dielectrics. Appl. Phys. Lett..

[11] Fortunato E, Barquinha P, Martins R (2012). Oxide Semiconductor Thin-Film Transistors: A Review of Recent Advances. Advanced Materials.

[12] Choi H-S, Jeon S (2015). Anomalous high photoconductivity in short channel indium-zinc-oxide photo-transistors. Appl. Phys. Lett..

[13] Ahn S (2015). Photoresponse of an oxide semiconductor photosensor. J. Vac. Sci. Technol. B.

[14] Lee E (2014). Nanocrystalline ZnON; High mobility and low band gap semiconductor material for high performance switch transistor and image sensor application. Sci. Rep..

[15] Choi H-S, Jeon S (2014). Thickness dependent low-frequency noise characteristics of a-InZnO thin-film transistors under light illumination. Appl. Phys. Lett..

[16] Heo J (2013). Graphene and thin-film semiconductor heterojunction transistors integrated on wafer scale for low-power electronics. Nano Letters.

[17] Jeon S (2011). Short channel device performance of a-InGaZnO thin film transistor. Appl. Phys. Lett..

[18] Kim, S. I. *et al*. High Performance Oxide Thin Film Transistors with Double layers. *IEDM* **1** (2008).

[19] Yu X (2013). A synergistic approach to high-performance oxide thin film transistors using a bilayer channel architecture. ACS Appl. Mater. Interfaces.

[20] Park JC (2010). Highly Stable Transparent Amorphous Oxide Semiconductor Thin-film Transistors Having Double-stacked Active layer. Advanced Materials.

[21] Munzenrieder N (2013). Flexible Self-Aligned Amorphous InGaZnO Thin-Film Transistors With Submicrometer Channel Length and a Transit Frequency of 135 MHz. IEEE Transaction on Electron Devices.

[22] Heo J (2013). Graphene and thin-film semiconductor heterojunction transistors integrated on wafer scale for low-power electronics. Nano Letters.

[23] Choi H (2011). Verification of Interface State Properties of a-InGaZnO Thin Film Transistors with SiN_x_ and SiO_2_ gate dielectrics by low-frequency noise measurements. IEEE Electron Device Lett.

[24] Lee S (2011). Trap-limited and percolation conduction mechanisms in amorphous oxide semiconductor thin film transistor. Appl. Phys. Lett..

[25] Jeon S (2011). Nanometer Scale Oxide Thin Film Transistor for High Density Image Sensor Applications. ACS Appl. Mater. Interfaces.

[26] Ghaffarzadeh K (2010). Persistent photoconductivity in Hf-In-Zn-O thin film transistors. Appl. Phys. Lett..

[27] Ghaffarzadeh K (2010). Instability in threshold voltage and sub-threshold behavior in Hf-In-Zn-O thin film transistors induced by bias- and light-Stress. Appl. Phys. Lett..

[28] Kim M-G, Kanatzidis MG, Facchetti A, Marks TJ (2011). Low-temperature fabrication of high-performance metal oxide thin-film electronics via combustion processing. Nature Materials.

[29] Jeon S (2011). Nanometer-scale oxide Thin Film Transistor with Potential for High Density Image Sensor Applications. ACS Appl. Mater. Interfaces..

[30] Jeon S (2011). Short channel device performance of amorphous InGaZnO thin film transistor. Appl. Phys. Lett..

[31] Seo D (2010). Fully transparent InGaZnO thin film transistors using indium tin oxide/graphene multilayer as source/drain electrodes. Appl. Phys. Lett..

[32] Choi H-S, Jeon S (2014). Field-induced macroscopic barrier model for persistent photoconductivity in nanocrystalline oxide thin-film transistors. Appl. Phys. Lett..

[33] Jeon S (2010). Low-frequency Noise Performance of a bilayer InZnO-InGaZnO Thin Film Transistor for Analog Device Applications. IEEE Electron Device Lett.

[34] Choi H (2011). Verification of interface state properties of a-InGaZnO thin-film transistors with and gate dielectrics by low frequency noise measurements. IEEE Electron Device Lett..

[35] Cheong WS (2012). Current stress induced electrical instability in transparent zinc tin oxide thin-film transistors. J. Nanosci. Nanotechnology.

[36] Bersuker G (2007). Mechanism of Electron Trapping and Characteristics of Traps in HfO2 Gate Stacks. IEEE Trans. Dev. Mat. Reliability.

[37] Young CD (2009). Pulsed–methodology and its application to electron trapping characterization and defect density profiling. IEEE Trans. Electron. Device Lett..

[38] Ahn S (2013). High-performance nanowire oxide photo‐thin film transistor. Advanced Materials.

[39] Park JC, Kim SW, Kim CJ, Lee H-N (2012). The Effects of Gadolinium Incorporation Into Indium–Zinc–Oxide Thin-Film Transistors. IEEE Electron Device Letters.

[40] Barquinha P, Pimental A, Marques A, Pereira L, Martins R, Fortunato E (2006). Influence of the semiconductor on the electrical properties of transparent TFTs based on Indium Zinc Oxide. J. Non-Cryst. Solids.

[41] Itagaki N (2008). Zn-In-O based Thin-Film Transistors-compositional dependence. Phys. Status solidi A.

[42] Young CD (2005). Ultra-short pulse current–voltage characterization of the intrinsic characteristics of high-k devices. Japan. J. Appl. Phys..

[43] Lee Y (2011). Fast transient charging at the Graphene/SiO_2_ interface causing hysteretic device characteristics. Appl. Phys. Lett..

[44] Shen C (2006). A fast measurement technique of MOSFET Id–Vg characteristics. IEEE Electron Device Lett..

[45] Lee Y (2013). Quantitative analysis of hysteretic reaction at the interface of Graphene and SiO_2_ using the short pulse I-V method. Carbon..

[46] Young CD (2010). The pulsed Id–Vg methodology and its application to the electron trapping characterization of high-k gate dielectrics. J. Semicond. Technol. Sci..

[47] Woo H (2017). Determination of intrinsic mobility of bilayer oxide thin-film transistor by pulsed I-V method. Nanotechnology.

[48] Kim T (2016). Effect of hydrogen on dynamic charge transport in amorphous oxide thin film transistors. Nanotechnology.

[49] Lee E (2016). High mobility and high stability glassy metal-oxynitride materials and devices. Sci. Rep..

[50] Kim T (2015). Fast transient charging behavior of HfInZnO thin-film transistor. Appl. Physic. Lett..

[51] Heh D, Young CD, Bersuker G (2008). Experimental evidence of the fast and slow charge trapping/detrapping processes in high-k dielectrics subjected to PBTI stress. IEEE Electron Device Lett..

[52] Heh, D., Choi, R., Young, C. D. & Bersuker, G. Fast and slow charge trapping/detrapping processes in high-*κ* nMOSFETs. *In**Proc. IEEE IRW Final Report* 120–124 (2006).

[53] Kim T (2016). The influence of interfacial defects on fast charge trapping in Nano-crystalline oxide semiconductor thin film transistor. Semicond. Sci. Technol..

[54] Kim T (2017). Influence of fast charging on accuracy of mobility in a-InHfZnO Thin film transistor. IEEE Electron Device Lett..

[55] Paulsen RE, White MH (1994). Theory and application of charge pumping for the characterization of Si-SiO2 interface and near-interface oxide traps. IEEE Trans. Electron. Device.

[56] Groeseneken G, Maes HE, Beltran N, De Keersmaecher RF (1984). Reliable approach to charge-pumping measurements in MOS transistors. IEEE Trans. Electron Device.

[57] Jung U (2014). Quantitatively estimating defects in graphene device using discharge current analysis method. Sci. Rep..

[58] Jung U (2014). Quantitative analysis of interfacial reaction at a graphene/SiO_2_ interface using the discharging current analysis method. Appl. Physic. Lett..

[59] Jung U (2015). Extraction of the interface state density of top-gate graphene field-effect transistor. IEEE Electron Device Lett..

[60] Choi H (2012). The impact of active layer thickness on low-frequency noise characteristics in InZnO thin-film transistors with high mobility. Appl. Phys. Lett..

[61] Barquinha P, Goncalves G, Pereira L, Martins R, Fortunato E (2007). Effect of annealing temperature on the properties of IZO films and IZO based transparent TFTs. Thin Solid Films.

[62] Kim T (2016). Pulse I-V characterization of a Nano-crystalline oxide device with sub-gap density of states. Nanotechnology.

[63] Lee S (2010). Extraction of subgap density of states in amorphous InGaZnO thin-film transistors by using multifrequency capacitance–voltage characteristics. IEEE Electron Device Letters.

[64] Chen C, Abe K, Kumomi H, Kanicki J (2009). Density of states of a-InGaZnO from temperature-dependent field-effect studies. IEEE Trans. Electron Devices.

[65] Jeon YW (2010). Sub-gap density of state based amorphous oxide thin film transistor simulator(DeAOTS). IEEE Trans. Electron. Device..

[66] Lee S (2015). Oxygen defect-induced metastability in oxide semiconductors probed by gate pulse spectroscopy. Sci. Rep..

[67] Lee E (2015). Ar plasma treated ZnON transistor for future thin film electronics. Appl. Physic. Lett..

[68] Ao L (2013). High-performance InTiZnO thin-film transistors deposited by magnetron sputtering. Chin. Phys. Lett..

[69] Kim T (2016). The influence of Hydrogen on defects of In-Ga-Zn-O semiconductor thin-film transistors with atomic-layer deposition of Al_2_O_3_. IEEE Electron Device Letters.

